# CAR-T Cell Therapy for Autoimmune Kidney Diseases: Where Do We Stand Now?

**DOI:** 10.3390/ijms262010070

**Published:** 2025-10-16

**Authors:** Beata Kaleta

**Affiliations:** Department of Clinical Immunology, Medical University of Warsaw, Nowogrodzka 59, 02-006 Warsaw, Poland; beata.kaleta@wum.edu.pl

**Keywords:** autoimmune kidney disease, chimeric antigen receptor, immunotherapy

## Abstract

Autoimmune kidney diseases (AIKDs) are a consequence of the dysregulation of immune response and the loss of tolerance to self-antigens, which led to glomerulonephritis and tissue damage. Autoantibody-producing B cells, as well as T cells, neutrophils and macrophages play a pivotal role in the pathogenesis and progression of various AIKDs. In recent years, B cell-depleting/modulating therapies and molecules that modulate T cell differentiation pathways and cytokine production have become a new hope for patients with immune-mediated kidney diseases. However, these biologicals often do not bring satisfactory therapeutic benefits, which is most likely related to incomplete B cell depletion of tissue-resident B cells. A new hope is immunotherapy with chimeric antigen receptor (CAR) effector cells. In CAR therapy, immune cells (mostly T cells) are genetically modified to express a CAR, which enables the recognition of the specific antigen on a target cell. This interaction leads to the formation of immune synapse and cytotoxicity. CAR-based strategies are a potent form of cell therapy that offers a better chance for deep and durable response than other recently approved immune therapies. Moreover, CAR-T cells can be programmed for higher precision and safety. This review explores the current landscape of CAR-T cell therapy in AIKDs.

## 1. Introduction

Autoimmunity is a consequence of the loss of central tolerance to self-antigens or impaired peripheral suppressive immune response regulation [1]. It is associated with the activation of autoreactive immune cells and inflammation, which cause pathological damage to various organs, including the kidneys [2]. Current data indicate that approximately 850 million people are affected by different types of chronic kidney disorder, including autoimmune kidney diseases (AIKDs) and their frequency varies greatly depending on the condition [3]. For example, the incidence of membranous nephropathy (MN) is 8–10 cases per 1 million population per year, focal segmental glomerulosclerosis (FSGS) 7–8 cases, antiglomerular base membrane (anti-GBM) nephropathy about 0.5–2 cases, antineutrophil cytoplasmic antibody (ANCA) associated vasculitis (AAV) 17.2 cases, while lupus nephritis (LN) 0.7–1.3 cases per 100,000, IgA nephropathy (IgAN) 2.5 cases per 100,000 and minimal change disease (MCD) 2–7 cases per 100,000 children and 0.6 cases per 100,000 adults [2,3,4].

AIKDs pathogenesis is multifactorial and involves an interplay of genetic predispositions, environmental factors and the dysregulation of the immune system, which leads to inflammation, tissue damage, and kidney failure [2,4].

Currently, immune-mediated glomerulopathies are treated with corticosteroids alone or by the combination of corticosteroids with immunosuppressive agents, which is frequently associated with serious side effects or loss of response to the therapy [2,3]. Therefore, in recent years, considerable research efforts have been made to develop novel AIKDs therapeutic strategies, including adoptive cell therapy (ACT) with chimeric antigen receptor (CAR) redirected T cells [5]. These genetically engineered CAR-T cells recognize the surface antigen of target cells and selectively kill these cells [6]. Therefore, CAR-based therapies targeting cells associated with AIKDs pathogenesis have emerged as great therapeutic potential.

In this review, I describe the immunologic mechanisms associated with AIKDs, outline the evolution of CAR structure and mechanism of action, as well as focus on the therapeutic potential of various CAR-expressing effector cells in AIKDs. Moreover, I summarize findings from studies conducted on animal models and pre-clinical studies, as well as ongoing clinical investigations.

## 2. Pathogenic Mechanisms in AIKDs

The development of AIKDs is associated with the dysregulation of immune system homeostasis, which results from the interaction between genetic and environmental factors.

In direct AIKDs, antibodies (abs) target specific kidney antigens, while indirect AIKDs are a consequence of autoantibody-induced glomerular damage or glomerular immune complex deposition in the course of systemic disorders [7]. Examples of direct AIKDs include MN, FSGS, and anti-GBM nephropathy. In contrast, LN, IgAN, and AAV are classified as indirect AIKDs [7]. Another immune-mediated kidney disease is MCD, a podocytopathy that leads to nephrotic syndrome [8]. Autoantibodies targeting nephrin (an essential structural component of the podocyte slit-diaphragms) have been found in patients with MCD, however, the disease pathophysiology is still obscure [9].

**Figure 1:**
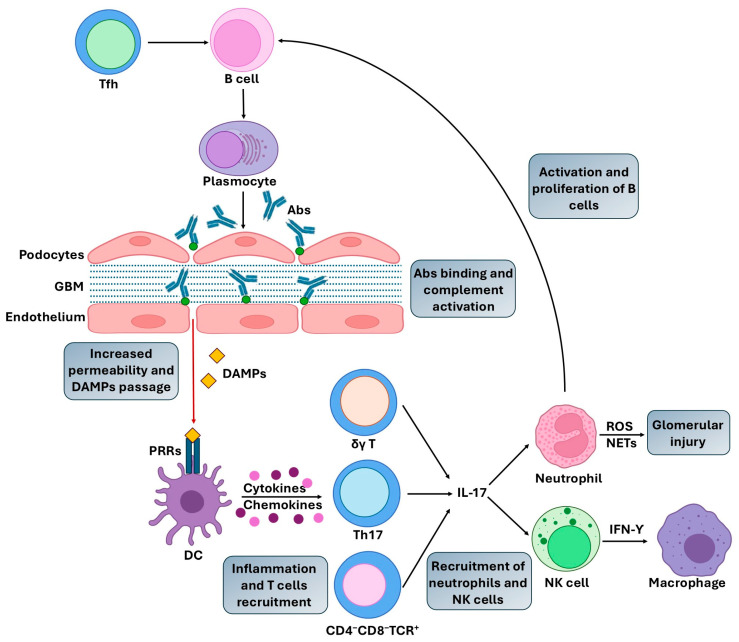
Pathogenic mechanisms in autoimmune kidney diseases. Antibodies (Abs) produced by plasmocytes, plasmablasts, and memory B cells bind to renal antigens or nonspecific circulating autoantigens and are deposited in glomeruli as immune complexes. Immune complexes deposition and complement activation lead to increased permeability and passage of damage-associated molecular patterns (DAMPs). DAMPs bind and activate pattern recognition receptors (PRRs) which leads to the production of proinflammatory cytokines and chemokines, and the activation of interleukin (IL)-17-producing T-helper (Th)-17 cells, γδ T cells, and CD4−CD8−TCR+ (T cell receptor) T cells. IL-17 promotes the recruitment of neutrophils and natural killer (NK) cells. Neutrophils secrete reactive oxygen species (ROS) and neutrophil extracellular traps (NETs) that contribute to glomerular injury. NK cells secrete interferon-gamma (IFN-γ), which promotes macrophages activation, resulting in the progression of renal inflammation. DC, dendritic cell; GBM, glomerular basement membrane; Tfh, T follicular helper cells.

As mentioned above, the pathogenic mechanisms in AIKDs involve the production of autoantibodies that bind various target renal antigens or nonspecific circulating autoantigens deposited in glomeruli, the activation of T cells, macrophages, dendritic cells and neutrophils, and the secretion of immuno-modulatory cytokines and chemokines [2,10,11] (Figure 1). Mature B cells differentiate into antibodies (abs)-secreting plasma cells, plasmablasts, as well as memory B cells [12]. Autoantibody-mediated renal damage is associated with the formation of immune complexes, which are generated by the binding of abs to antigens, and their deposition in the renal glomeruli. Immune complexes deposition and subsequent complement activation lead to increased permeability and the passage of low molecular weight molecules, as well as damage-associated molecular patterns (DAMPs) release. DAMPs are endogenous molecules released from damaged, stressed, or necrotic cells. They represent a large range of mediators that bind and activate pattern recognition receptors (PRRs) on dendritic cells (DCs) or kidney cells, which leads to a cascade of renal injury [13]. Activation of DAMP-sensing receptors induces the production of proinflammatory cytokines and chemokines, and the recruitment and activation of various populations of interleukin(IL)-17-producing T cells, including T-helper (Th)-17 cells, γδ T cells, and CD4−CD8−TCR+ (T cell receptor) double negative T cells. IL-17 plays a crucial role in recruiting neutrophils and, indirectly, natural killer (NK) cells, as well as promoting B cell maturation and autoantibody production, which leads to kidney disfunction [13,14]. Infiltrated neutrophils within the glomerulus secrete a variety of substances that contribute to glomerular injury, such as reactive oxygen species (ROS), proteases, and neutrophil extracellular traps (NETs), among others [15]. In turn, infiltrated NK cells are a key source of interferon-gamma (IFN-γ), which promotes macrophage activation, resulting in the progression of renal inflammation [16].

Several approaches have been proposed to target the above-mentioned immune cells in AIKDs therapy, including B cell-depleting/modulating therapies and molecules that modulate T cells differentiation pathways and cytokines production.

Notwithstanding advancements made in the field of diagnosis and treatment of AIKDs, numerous patients do not achieve an adequate response or suffer from serious adverse effects of standard immunosuppressive therapy. Therefore, novel treatment strategies, which offer a better chance for deep and durable response, are actively being researched and developed.

In recent years, adoptive cell therapy (ACT) using chimeric antigen receptor (CAR)-expressing cells has shown great promise in the treatment of a range of hematological malignancies [17]. Moreover, the use of CAR-based therapy has been investigated in some inflammatory and autoimmune diseases in the preliminary stages of clinical research [18].

## 3. CAR Structure and Generations

**Figure 2:**
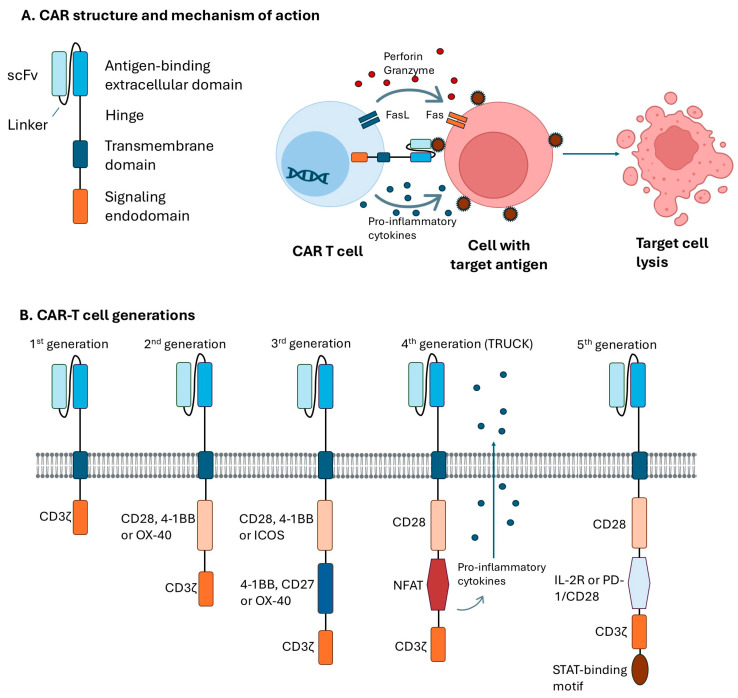
Schematic representation of the structure and generations of chimeric antigen receptors (CARs). (**A**) CAR structure and mechanism of action. (**B**) CAR-T cell generations.

In CAR-based immunotherapy, effector immune cells, mostly T cells, but also NK cells or macrophages, are genetically modified to express CARs, fusion proteins designed to recognize, target, and eliminate cells with specific surface antigens [19,20]. CARs general structure consists of an extracellular antigen-binding domain, transmembrane domain (serving as the anchor to the effector cell membrane), and signaling endodomain that activates the effector cell upon binding to the target antigen [21]. Currently, CAR-T cells are classified into five generations depending on how their intracellular signaling domain is organized (Figure 2).

First-generation CAR constructs contain a single-chain variable fragment (scFv), a hinge domain/spacer, and an intracellular CD3ζ signaling domain, without costimulatory domains. The clinical value of these CARs was limited, which was associated with the low proliferation and persistence of modified effector cells. Second-generation CARs contain additional cytoplasmic costimulatory domains such as CD28, 4-1BB, or OX-40, which provide their improved proliferation, persistence, and higher cytotoxicity. Third-generation CARs include two costimulatory domains (CD28, 4-1BB, or ICOS and 4-1BB, CD27, or OX-40) which is associated with their greater expansion and longer persistence compared to second-generation. Fourth-generation CARs are also named T cells redirected for antigen-unrestricted cytokine-initiated killing (TRUCKs) CARs. TRUCK-CAR-T cells are transduced with cassette for the nuclear factor of activated T-cells (NFAT)-mediated transgene expression, and thus are able to recruit other immune cells via a CAR-inducible transgenic immunomodulator, such as IL-12, IL-18, and IL-23, to enhance antitumor response. Fifth-generation CARs are currently developed for a better safety profile and a more comprehensive therapeutic effect. They are being equipped with various membrane receptors, to recognize more than one antigen or low antigen density targets. For example, these CARs may have integrated receptor for IL-2 (IL-2R), or programmed cell death protein 1 (PD-1)/CD28 switch-receptor, with a STAT-binding motif for JAK/STAT signaling [21,22,23].

Currently, there are six commercial CAR-T products approved by the by the Food and Drug Administration (FDA) and European Medicines Agency (EMA): tisagenlecleucel (Kymriah^®^), axicabtagene ciloleucel (Yescarta^®^), brexucabtagene autoleucel (Tecartus^®^), lisocabtagene maraleucel (Breyanzi^®^), idecabtagene vicleucel (Abecma^®^), and relmacabtagene (Relma-cel^®^) [24]. All these products are second-generation CAR-T cell therapies.

## 4. Current B-Cell Therapy for AIKDs

B-cell targeted therapy (either B cell depletion or B cell modulation) has been approved by FDA for the treatment of a wide spectrum of autoimmune diseases, including some AIKDs. Autoreactive B cells play a key role in the pathogenesis of immune-mediated diseases, therefore direct or indirect B cells depletion with monoclonal antibodies (mAbs), inhibition of B cells activation or co-stimulatory blockade are the main strategies used in the treatment of AIKDs.

Rituximab, the anti-CD20 mAb effectively depletes CD20-positive B cells, but, do not affect CD20-negative pro-B cells and plasmablasts [25]. Although the drug is used in LN, AVV and MN, it does not lead to a complete response in some patients, therefore, the next generation anti-CD20 mAb has been developed: ocrelizumab and obinutuzumab. These mAbs have higher receptor affinity and enhanced antibody-dependent cytotoxicity. Due to high rate serious infections, the clinical trial with the ocrelizumab (NCT00626197) has been suspended [26], however, obinutuzumab used in combination with mycophenolate mofetil plus oral prednisone led to better clinically meaningful improvements in the percentage of patients with a complete renal response, as compared with placebo or standard treatment (NCT04221477) [27]. Inebilizumab, an anti-CD19 mAb, and blinatumomab, a bispecific T-cell engager (BiTE) Ab that targets both CD19 and CD3 are currently under evaluation in a clinical trial (NCT06570798) with adult patients with LN [28]. In addition, two anti-CD38 mAbs, daratumumab and felzartamab, are also used in the treatment AIKDs [29]. CD38 antigen is expressed on several immune cells, including plasma cells. Daratumumab has been utilized successfully in patients with MN, LN, and refractory AAV. In turn, felzartamab decreased proteinuria and improved estimated glomerular filtration rate (eGFR) in patients with IgAN [30].

In addition to the above-mentioned B cell-depleting drugs, several B cells-modulating agents have been used in the treatment of various AIKDs. Abs targeting B-cell activating factor (BAFF) and proliferation-inducing ligand (APRIL) have emerged as a potential therapeutic option in the management of IgAN. BAFF and APRIL are members of the tumor necrosis factor (TNF) superfamily, which bind to B-cell maturation antigen (BCMA, expressed on plasma cells), transmembrane activator, calcium-modulator, cytophilin ligand interactor (TACI, on mature B cells and plasma cells), and BAFF receptor (BAFF-R, expressed on immature and mature naive B cells). Both BAFF and APRIL play a crucial role in B cell activation, abs production, and immune complex formation [28]. The efficacy and safety of belimumab, a mAb targeting soluble BAFF, approved for systemic lupus erythematosus (SLE) therapy, was analyzed in patients with LN. It was found that belimumab used with standard treatment (cyclophosphamide, mycophenolate mofetil, azathioprine, or methotrexate) improved renal response rates and reduced the risk of renal function worsening [31]. However, it was revealed that this mAb did not reduce the risk of AAV recurrence (NCT03967925) [32]. In contrast, in phase II trial (NCT03949855) belimumab used with combination with rituximab in patients with primary MN lead to reduced proteinuria, and decreased production of anti-phospholipase A2 receptor (PLA2R) abs (target autoantigen in MN) when compared to rituximab alone.

Atacicept and telitacicept are both dual BAFF/APRIL inhibitors, and showed beneficial effects in patients with LN (NCT05609812) and IgAN (NCT04716231) [33]. These drugs effectively reduced proteinuria and improved kidney function. Similarly, decreased proteinuria was observed in IgAN patients treated with sibeprenlimab, an anti-APRIL mAb (NCT05248646) [34]. All these products are second-generation CAR-T cell therapies. Currently, no commercial products are first-generation CAR-T cells. Third to fifth-generation are still in development and are not approved for commercial use.

## 5. CAR-Based Therapy for AIKDs

CAR-based therapy has emerged as a revolutionary approach in the treatment of B cell hematological malignancies, including B cell lymphoma, B cell acute lymphoblastic leukemia, and multiple myeloma. Its potential application in autoimmune diseases is currently explored and has entered early-stages clinical trials [35]. B cell-targeting CAR T-cells offer a better chance for deep and durable response than other recently approved anti-B cell therapies. As a living drug, CAR cells have been shown to persist for more than a decade in humans [36,37]. Moreover, CAR cells are more effective at killing targeted cells at low antigen density than was the analogous abs, and importantly, they can be programmed by logic-gating for higher precision, strength, and safety [38,39]. Therefore, AIKDs are promising candidates for B cell targeting in CAR T-cell therapy.

### 5.1. Studies Conducted on Animal Models

As mentioned above, the administration of anti-CD20 abs did not bring the satisfactory therapeutic benefits to patients with LN, which was most likely related to the incomplete B cell depletion of tissue-resident B cells. Therefore, Kansal and colleagues [40] investigated whether anti-CD19 CAR T cells effectively and permanently depleted B cells in a mouse model of LN. The group transduced splenic CD8+ T cells to express anti-CD19 CAR with CD28 and CD3ζ signaling domains and injected 1.2 × 10^6^ effector cells per NZB/W and MRL-lpr mice. It was revealed that these anti-CD19 CAR CD8+ T cells permanently eliminated CD19+ B cells and effectively decreased total serum IgM and IgG, as well as anti-DNA IgG and IgM autoantibodies. Moreover, CARs administration significantly reduced proteinuria, and increased the lifespans of both lupus mice. It was also observed that CD19+ B cell depletion reduced splenomegaly, immune cells infiltration in glomeruli, and skin lesions. It is worth emphasizing that normal splenic B cells and bone marrow-resident plasma cells were not depleted by examined anti-CD19 CAR-T cells. Later analysis showed that T cells transduced with this second-generation CAR were active for one year in vivo.

In a similar study conducted by Jin et al. [41], 293F T cells have been transduced to express a second-generation anti-CD19 CAR with 4-1BB or CD28 signaling domain and evaluated in murine SLE model with severe LN. MLR-lpr mice were injected with 1.005 × 10^6^ effector cells and CD19+ B cell depletion efficiency was analyzed. It was revealed that, 7 days after the treatment, CD19 CAR-T cells preceded by body irradiation depleted almost all circulating CD19+ B cells in the blood and the effect lasted 9 weeks after transfer. Moreover, the number of CD19−CD138+ plasma cells was also decreased. CAR therapy extend lifespan of all mice, reduced spleen weight and ameliorated skin symptoms. In addition, preconditioned animals receiving CAR-T cell showed mild glomerulonephritis, with lower infiltration of lymphocytes and immune complex deposition. However, in contrast to Kansal et al. [38], the group did not observe decreased proteinuria, serum anti-dsDNA and antinuclear abs concentrations reduction. In a recent study [42], the efficacy of CD19 CAR T cells in a murine myeloperoxidase (MPO)-AVV model was examined. The second-generation CD19 CAR with CD28 costimulatory domain was constructed and injected to mice (2 × 10^6^ CAR T cells). It was found that up to 8 weeks after transfer, CAR-T cells engrafted in spleen, bone marrow, lymph nodes, peripheral blood and kidneys and significantly reduced the number of CD19 B cells, plasmablasts and intermediate plasma cells, but not plasma cells in these compartments. Moreover, CD19 CAR-T cell treatment reduced albuminuria and urinary neutrophil gelatinase-associated lipocalin (marker of kidney damage), as well as MPO-anti-neutrophil cytoplasmic autoantibody (ANCA) abs concentration. In addition, the number of kidney-infiltrating neutrophils and lymphocytes were reduced, however, no significant differences were observed for monocytes, eosinophils, erythrocytes, hemoglobin, and hematocrit.

### 5.2. Pre-Clinical and Clinical Investigations

The promising results of mentioned above pre-clinical studies on animal models, as well as results of analyses conducted in patients with various autoimmune diseases were essential for advancing research into clinical trials of CAR-T cell therapy for AIKDs.

Several studies conducted on patients with SLE and LN demonstrated that B cell depletion with anti-CD19 CAR-T cells resulted in the improvement of kidney function. Mougiakakos et al. [43] administered 1.1 × 10^6^ per kg body weight of autologous anti-CD19 CAR-T cells in a female patient with severe and refractory SLE with active LN and nephrotic syndrome. It was revealed that, after the injection, the number of CAR-T cell rapidly increased, and these cells were detectable for 7 weeks. Anti-CD19 CAR-T cell treatment resulted in complete depletion of circulating B cells, significant reduction of a double-stranded DNA (dsDNA) autoantibodies within 5 weeks, and normalization in complement factor C3 and C4 levels. Moreover, a decrease in proteinuria and disease activity was observed. It is worth emphasizing that the patient did not have any adverse events associated with the CAR therapy and there were no relapses during 18 months of follow-up. In the next study conducted by the same group, five patients with active SLE were treated with autologous anti-CD19 CAR-T cells (single infusion at dose of 1 × 10^6^ CAR-T cells per kg body weight) [44]. After the administration, a rapid expansion of the CAR-T cells was observed in all patients which resulted in a significant depletion of B cells. Similar to a previous study, a remission of SLE was achieved in all patients after 3 months and was maintained 12 months after the treatment. In all patients, B cell reconstitution was observed after an average time of 110 ± 32 days, however, these B cells were naïve and showed non-class-switched B cell receptors and no relapse of SLE was observed in the long-term follow-up. Histologic analysis revealed podocyte damage, but immune complex deposition, complement activation, and lupus nephritis were not observed. The treatment was well tolerated with only mild cytokine release syndrome (CRS). What is interesting is that the group also analyzed CAR-T cell therapy’s impact on abs titers of vaccinations carried out before the treatment and demonstrated no decrease of vaccination responses.

Müller and colleagues [45] evaluated the effects of anti-CD19 CAR-T cell therapy in a group of patients with various autoimmune diseases, including eight patients with LN. It was revealed that after single infusion of 1 × 10^6^ autologous CAR-T cells per kg body weight, modified cells rapidly expanded in vivo and eliminated CD19 B cells. The treatment decreased disease activity, eliminated anti-dsDNA abs, normalized complement factor C3 levels, and reduced proteinuria. Similar to Mackensen et al. [44], stable levels of vaccine-associated abs were observed after 29 months after the adoptive transfer of CD19-targeted CAR-T cells.

Anti-CD19 CAR-T cells therapy has shown benefits in pediatric (15-year-old) patient with severe and rapidly progressive SLE with LN [46]. 1 × 10^6^ anti-CD19 CAR-T cells per kg body weight were infused and led to complete depletion of B cells, which persisted 6 months after the end of treatment. No serious toxic effects were observed, except for mild CRS. CAR-T cells administration resulted in decreased disease activity, complement factor C3 and C4 normalization, and depletion of anti-dsDNA and other autoantibodies. Moreover, decreased creatinine level, increased eGFR and decreased proteinuria were observed. The patient no longer required hemodialysis (the last took place on day 17).

In a similar study, ref. [47] two pediatric patients (both 12-year-old) with active SLE and LN have been treated with autologous anti-CD19 CAR-T cells (1 × 10^5^ cells per kg body weight). Both patients developed mild CRS. One patient developed mild immune effector cell-associated neurotoxicity syndrome (ICANS). In addition, in both patients the disease activity decreased. Laboratory tests showed decreased IgG concentrations, significantly reduced proteinuria, and complement C3 and anti-ds DNA abs levels normalization.

These promising results obtained in LN patients prompted the initiation of a several clinical studies the aim of which is to assess the safety and effectiveness of various CAR-expressing effector cells in AIKDs (summarized in Table 1).

## 6. Challenges of CAR-T Therapy in AIKDs

CAR-based therapy holds great promise for the treatment of autoimmune diseases. In light of the growing interest of this immunotherapy in AIKDs, it is imperative to comprehensively elucidate the underlying pathophysiological mechanisms driving treatment-related toxicity, and identify risk factors associated with its development. Other critical challenges associated with this therapy are effectiveness, stability, as well as accessibility. Another concern is manufacturing, which is expected to overcome mentioned above limitations. Therefore, identification of potential patients and therapeutic targets is indispensable to make a decision about implementing CAR therapy in patients with immune-mediated nephropathies.

The most severe prevalent toxicity in CAR-T cell therapy is a cytokine release syndrome (CRS) [48]. It is a result of activation and proliferation of CAR-T cells, which release proinflammatory cytokines (including TNF-α, IFN-γ, IL-6, IL-1β, and GM-CSF), which, in turn, activates monocytes and macrophages and consequently cause a cytokine storm and organ damage [49]. Another common toxicity associated with CAR treatment is immune effector cell-associated neurotoxicity syndrome (ICANS), which often occurs after CRS [50]. The mechanism associated with ICANS is not fully understood, however, it is believed to be related to the secretion of proinflammatory cytokines by monocytes and macrophages, increasing vascular permeability and the disintegration of the blood–brain barrier [49,50]. Another toxicity associated with CAR therapy, which may occur during the first days post-infusion, is acute kidney injury (AKI). It is a common but mild complication associated with release of inflammatory mediators, fluid loss, electrolyte abnormalities, and reduction in effective blood volume [51,52]. According to the latest reports, AKI may affect 5 to 30% of patients treated with CAR-T cells, especially with a history of chronic kidney disease or anticancer drug nephrotoxicity [53]. Although current research results suggest that both, CRS and ICANS are less common in patients with autoimmune diseases than in cancer patients, and can be effectively treated, these toxic effects of CAR-based therapy must be taken into account when a treatment plan is developed.

Beyond toxicity, appropriate candidates and target selection are the most fundamental factors determining the potential of CAR therapy of immune-mediated kidney disorders. AIKDs have very diverse nature, therefore, precise immunophenotyping and target antigens identification are crucial to reduce the risk of off target effects and organ damage.

To reduce CARs toxicity and enhance their clinical efficacy, various strategies are used during the designing, manufacturing, and using of CAR-T cells. Modification of an antigen binding domain can enhance CAR-T cells’ sensitivity to low-antigen-density or reduced toxicity [54]. Incorporation of multiple scFvs, in turn, enables the targeting of several antigens simultaneously. Recent studies have demonstrated that targeting receptors expressed on B cells or plasmocytes (e.g., BAFF-R, TACI and BCMA) more effectively eliminates autoreactive B cells, long-lived plasma cells, and downregulates autoantibodies production and proteinuria in SLE patients [55]. To reduce toxicity, “off-switches” or suicide genes can be incorporated. These modifications enable rapid elimination of CAR-T cells in response to adverse reactions. Several suicide safety switch mechanisms have been explored, including inducible caspase 9, FoxP3 inhibitors or modification of hinge and transmembrane domain to downregulate cytokine production [56].

Long-term adverse effects and manufacturing costs reduction can be achieved by nonviral delivery systems, including mRNA-based technologies and nanoparticles. These systems enable the production of functional and stable CAR-T cells with lower toxicity and improved efficacy (prolonged persistence). Moreover, using these systems offers multiple manufacturing benefits, including reduction of time and costs of CAR-T cells production. CAR-T therapy with in vivo-generated cells is another potential solution to complicated and expensive manufacturing. The in vivo-induced CAR-T cells production offers the possibility of product standardization, reduced risk of the most common toxicities, no need for preconditioning lympho-depleting chemotherapy, and a lower risk of resistance to CAR-T cell treatment [20].

## 7. Conclusions

The application of CAR-based therapy to AIKDs is a significant advancement in immunotherapeutic strategies. Genetically modified CAR-T or CAR-NK cells able to target and very specifically eliminate cells crucial for immune-mediated glomerulopathies offers a better chance for deep and durable response than other recently approved therapies.

Existing data from pre-clinical studies suggest that the application of CAR-T cell therapy to AIKDs could represent a significant advancement in therapeutic strategies. This therapy holds promise in treating these diseases, in particular LN, by elimination of CD19 and CD20 B cells and reduction in autoantibodies production. However, several challenges remain. Thus, clinical studies coupled with technological innovations are urgently needed to assess the long-term efficacy and safety of CAR T cells in patients with AIKDs.

## Figures and Tables

**Table 1:** Ongoing clinical trials of CAR-based therapy for various autoimmune kidney diseases.

Clinical Trial ID	Target Disease	Study Design	Cell Source and Type	Status	Duration	Results
CD19						
NCT05938725	LN *	Phase I/II N = 32	Autologous anti-CD19 CAR-T cell	Recruiting	2022–2029	No
NCT05930314	LN	Phase I N = 12	Autologous anti-CD19 CAR-T cell	Enrolling by invitation	2023–2025	No
NCT05798117	LN	Phase I/II N = 24	Autologous anti-CD19 CAR-T cell	Not yet recruiting	2023–2026	No
NCT06121297	LN	Phase I/II N = 12	Autologous anti-CD19 CAR-T cell	Recruiting	2024–2027	No
NCT06581198	LN	Phase II N = 144	Autologous anti-CD19 CAR-T cell	Recruiting	2024–2033	No
NCT06585514	LN	Phase I/II N = 18	Autologous anti-CD19 CAR-T cell	Recruiting	2024–2025	No
NCT06152172	LN and AVV	Phase I N = 24	Autologous anti-CD19 CAR-T cell	Not yet recruiting	2024–2026	No
NCT06544330	LN	Phase I N = 48	Autologous anti-CD19 CAR-T cell	Recruiting	2025–2041	No
NCT06294236	LN and AVV	Phase I N = 36	Allogeneic anti-CD19 CAR-T cell	Recruiting	2024–2028	No
NCT06557265	LN	Phase I N = 48	Allogeneic anti-CD19 CAR-NK cell	Recruiting	2024–2027	No
NCT05859997	AVV	Not Applicable N = 15	Allogeneic anti-CD19 CAR-T cell	Enrolling by invitation	2023–2025	No
NCT06056921	AVV	Phase I N = 24	Autologous anti-CD19 CAR-T cell	Recruiting	2023–2026	No
NCT06685042	AVV	Phase I/II N = 8	Autologous anti-CD19 CAR-T cell	Recruiting	2024–2025	No
NCT06508346	AVV	Observational N = 12	Autologous anti-CD19 CAR-T cell	Recruiting	2024–2027	No
NCT06548607	AVV	Phase I N = 20	Autologous anti-CD19 CAR-T cell	Recruiting	2024–2027	No
NCT06549296	AVV	Phase I N = 12	Autologous anti-CD19 CAR-T cell	Recruiting	2024–2027	No
NCT06548620	AVV	Phase I N = 18	Autologous anti-CD19 CAR-T cell	Not yet Recruiting	2024–2027	No
NCT06420154	AVV	Phase I N = 9	Autologous anti-CD19 CAR-T cell	Not yet Recruiting	2024–2027	No
NCT06590545	AVV	Phase I/II N = 8	Autologous anti-CD19 CAR-T cell	Not yet Recruiting	2025–2027	No
NCT06690359	IgAN and MN	Phase I N = 12	Autologous anti-CD19 CAR-T cell	Not yet Recruiting	2024–2026	No
NCT06469190	Immune nephropathy	Phase I N = 36	Autologous anti-CD19 CAR-NK cell	Recruiting	2024–2026	No
CD20						
NCT06375993	LN	Phase I N = 180	Allogeneic anti-CD20 CAR-T cell	Recruiting	2024–2027	No
BCMA						
NCT06277427	LN and AVV	Not Applicable N = 24	Autologous anti-BCMA CAR-T cell	Recruiting	2024–2027	No
NCT06497387	LN	Phase I N = 30	Autologous anti-BCMA CAR-T cell	Recruiting	2024–2027	No
CD19/CD20						
NCT06708845	LN	Phase I N = 48	Autologous anti-CD19/CD20 CAR-T cell	Not yet recruiting	2025–2026	No
NCT06462144	AVV	Phase I N = 36	Autologous anti-CD19/CD20 CAR-T cell	Recruiting	2024–2026	No
CD19/BCMA						
NCT06350110	LN and AVV	Phase I/II N = 75	Autologous anti-CD19/BCMA CAR-T cell	Recruiting	2024–2025	No
NCT06497361	LN	Phase I N = 30	Autologous anti-CD19/BCMA CAR-T cell	Recruiting	2024–2028	No
NCT06681337	LN	Phase I N = 10	Allogeneic anti-CD19/BCMA CAR-T cell	Not yet recruiting	2024–2025	No
NCT06285279	LN, AVV and MN	Phase I N = 24	Autologous anti-CD19/BCMA CAR-T cell	Recruiting	2024–2028	No
NCT05085418	Immune nephropathy	Phase I N = 9	Autologous anti-CD19/BCMA CAR-T cell	Unknown status	2021–2024	No
CD19/CD20/CD22						
NCT06653556	LN	Phase I N = 34	Autologous anti-CD19/CD20/CD22 CAR-T cell	Recruiting	2025–2029	No
CD19/CD3E						
NCT06373081	LN and AVV	Not Applicable N = 6	Autologous anti-CD19/CD3E CAR-T cell	Recruiting	2024–2026	No
BCMA/CD70						
NCT06553898	MDR-SRNS	Phase I N = 18	Autologous anti-BCMA/CD70 CAR-T cell	Recruiting	2024–2027	No

* AVV, anti-neutrophil cytoplasmic antibody (ANCA)-associated vasculitis; CAR, chimeric antigen receptor; IgAN, IgA nephropathy; LN, lupus nephritis; MDR-SRNS, multi-drug resistant nephrotic syndrome; MN, membranous nephropathy; NK, natural killer.

## Data Availability

No new data were created or analyzed in this study. Data sharing is not applicable to this article.

## Abbreviations

The following abbreviations are used in this manuscript:

Abs	Antibodies
ACT	Adoptive cell therapy
AIKDs	Autoimmune kidney diseases
AKI	Acute kidney injury
ANCA	Antineutrophil cytoplasmic antibody
APRIL	A proliferation-inducing ligand
AVV	ANCA-associated vasculitis
BAFF	B-cell activating factor
CAR	Chimeric antigen receptor
CRS	Cytokine release syndrome
DAMPs	Damage-associated molecular patterns
DC	Dendritic cell
EMA	European Medicines Agency
FDA	Food and Drug Administration
FSGS	Focal segmental glomerulosclerosis
GBM	Glomerular basement membrane
GM-CSF	Granulocyte-macrophage colony stimulating factor
ICANs	Immune effector cell-associated neurotoxicity syndrome
IFN	Interferon
IgAN	Immunoglobulin A nephropathy
IL	Interleukin
LN	Lupus nephritis
MCD	Minimal Change disease
MN	Membranous nephropathy
MPO	Myeloperoxidase
NETs	Neutrophil extracellular traps
NFAT	Nuclear factor of activated T cells
NK	Natural killer
PD-1	Programmed death receptor 1
PRR	Pattern recognition receptor
ROS	Reactive oxygen species
scFv	Single-chain variable fragment
SLE	Systemic lupus erythematosus
TACI	Transmembrane activator and calcium-modulator and cytophilin ligand interactor
TCR	T cell receptor
Tfh	T follicular helper
Th	T helper
TNF	Tumor necrosis factor
TRUCKs	T-cells redirected for antigen-unrestricted cytokine-initiated killing
